# 2-Thio­ureido-1*H*-benzimidazol-3-ium chloride monohydrate

**DOI:** 10.1107/S1600536814006837

**Published:** 2014-04-16

**Authors:** C. N. Sundaresan, Dheeraj Kumar Singh, Jagadeesh Babu Nanubolu

**Affiliations:** a Department of Chemistry, Sri Sathya Sai Institute of Higher Learning, Brindavan Campus, Kadugodi, Bangalore 560 067, India; b X-ray Crystallography Division, Indian Institute of Chemical Technology, Hyderabad 500 007, India

## Abstract

In the title compound, C_8_H_9_N_4_S^+^·Cl^−^·H_2_O, the cation is approximately planar, with a dihedral angle of 7.71 (8)° between the mean planes of the benzo­imidazole ring system and the thio­urea unit. In the crystal, cations, anions and water molecules of crystallization are linked by O—H⋯Cl, N—H⋯O, N—H⋯Cl and N—H⋯S hydrogen bonds into a three-dimensional network. π–π stacking is observed between the benzene and imidazole rings of neighbouring mol­ecules, the centroid–centroid distance being 3.5774 (11) Å.

## Related literature   

For the synthesis and biological activity of benzimidazoles, see: Siva & Subhash (2011 ▶); Sharghi *et al.* (2008 ▶); Refaat (2010 ▶); Puratchikody *et al.* (2008 ▶); Achar *et al.* (2010 ▶); Starcevic *et al.* (2007 ▶). For hydrogen-bond classification, see: Jeffrey *et al.* (1985 ▶).
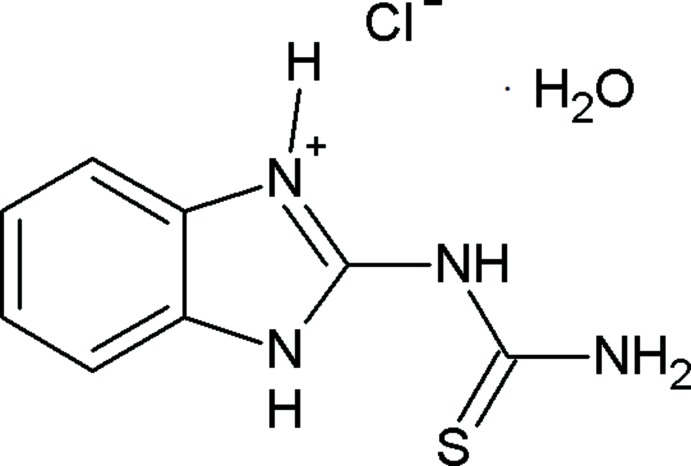


## Experimental   

### 

#### Crystal data  

C_8_H_9_N_4_S^+^·Cl^+^·H_2_O
*M* *_r_* = 246.72
Monoclinic, 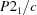

*a* = 9.3027 (6) Å
*b* = 8.7038 (5) Å
*c* = 14.5503 (8) Å
β = 109.143 (3)°
*V* = 1112.97 (11) Å^3^
*Z* = 4
Mo *K*α radiation
μ = 0.51 mm^−1^
*T* = 293 K
0.33 × 0.19 × 0.14 mm

#### Data collection  

Bruker SMART APEX CCD area-detector diffractometer
Absorption correction: multi-scan (*SADABS*; Bruker, 2001 ▶) *T*_min_ = 0.850, *T*_max_ = 0.932
10318 measured reflections
1958 independent reflections
1818 reflections with *I* > 2σ(*I*)
*R*_int_ = 0.021

#### Refinement  

*R*[*F* ^2^ > 2σ(*F* ^2^)] = 0.032
*wR*(*F* ^2^) = 0.083
*S* = 1.07
1958 reflections
136 parameters
H-atom parameters constrained
Δρ_max_ = 0.25 e Å^−3^
Δρ_min_ = −0.20 e Å^−3^

### 

Data collection: *SMART* (Bruker, 2007 ▶); cell refinement: *SAINT* (Bruker, 2007 ▶); data reduction: *SAINT*; program(s) used to solve structure: *SHELXS97* (Sheldrick, 2008 ▶); program(s) used to refine structure: *SHELXL97* (Sheldrick, 2008 ▶); molecular graphics: *ORTEP-3 for Windows* (Farrugia, 2012 ▶) and *Mercury* (Macrae *et al.*, 2008 ▶); software used to prepare material for publication: *publCIF* (Westrip, 2010 ▶).

## Supplementary Material

Crystal structure: contains datablock(s) I, New_Global_Publ_Block. DOI: 10.1107/S1600536814006837/xu5781sup1.cif

Structure factors: contains datablock(s) I. DOI: 10.1107/S1600536814006837/xu5781Isup2.hkl

Supporting information file. DOI: 10.1107/S1600536814006837/xu5781Isup3.cml

CCDC reference: 994075

Additional supporting information:  crystallographic information; 3D view; checkCIF report

## Figures and Tables

**Table 1:** Hydrogen-bond geometry (Å, °)

*D*—H⋯*A*	*D*—H	H⋯*A*	*D*⋯*A*	*D*—H⋯*A*
O1—H1*O*⋯Cl1^i^	0.81	2.29	3.0991 (15)	179
O1—H2*O*⋯Cl1	0.87	2.30	3.1443 (14)	165
N1—H1*N*⋯O1	0.86	2.32	3.064 (2)	145
N1—H2*N*⋯O1^ii^	0.86	2.14	3.000 (2)	174
N2—H2⋯O1	0.86	2.03	2.8667 (19)	165
N3—H3*N*⋯S1	0.80	2.43	3.0146 (14)	131
N4—H4*N*⋯Cl1	0.83	2.28	3.1055 (15)	175

Symmetry codes: (i) 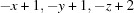; (ii) 
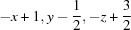
.

**Fig. 1.**
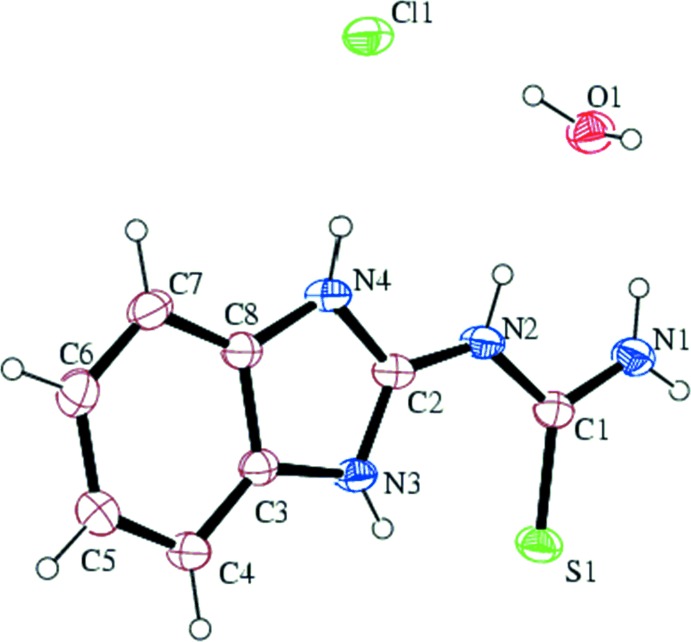
ORTEP diagram of 1-(1H-benzo[d]imidazol-2-yl)thiourea salt hydrate (Thermal ellipsoids are drawn at 30% probability level).

**Fig. 2.**
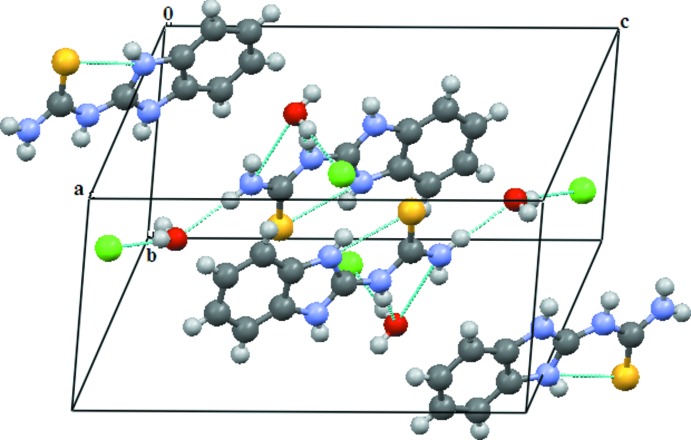
Crystal packing diagram of the title compound (I).

**Fig. 3.**
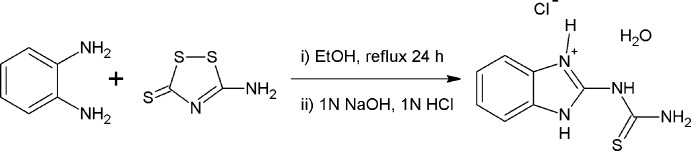
Synthetic scheme of the title compound (I).

## supplementary crystallographic information

### 1. Comment

In recent years, Benzimidazole moiety has gained increased interest in drug industry worldwide, as an important pharmocophore exhibiting a wide spectrum of biological and pharmaceutical activities. They act as anti-HIV agents, anti cancer agents (Refaat, 2010), anti-tumor agents (Starcevic *et al.*, 2007), anti-microbial agents (Puratchikody *et al.*, 2008) analgesic and anti-inflammatory agents (Achar *et al.*, 2010).

The title compound (Fig. 1), C_8_H_11_ClN_4_OS, crystallized in monoclinic *P*2_1_/*c* space group with *Z*=4 (Fig. 2). Two chloride anions and two water molecules are acting as a bridge to connect four molecules of the title compound which resulted in infinite layered type supramolecular architecture. The title compound is mainly stabilized by N—H···O, N—H···Cl, O—H···Cl intermolecular hydrogen bonding which resulted in generating of ring motifs *R*^2^_2_ (8) and *R*^1^_2_ (6). The intramolecular ring motif *S*^1^_1_ (6) is also generated due to intramolecular N—H···S hydrogen bonding.

### 2. Experimental

A mixture of 5-amino-3*H*-1, 2, 4-dithiazole-3-thione (1.5 g, 0.01 mol) and *o*-phenylenediamine (1.08 g, 0.01 mol) in absolute ethanol (25 ml) was refluxed for 24 h. The solvent was removed under reduced pressure and the residue was treated with aqueous sodium hydroxide (1 N, 3 × 20 ml) and then filtered after an hour. The filtrate was adjusted to pH 5 by addition of aqueous hydrochloric acid (1 N) and left in a refrigerator overnight. The precipitate (1.8 g, 74%) was collected, washed with water and dried. The title compound was recrystallized from formic acid-propanol mixture to yield small crystals. The melting point was recorded as 248–251°C.

### 3. Refinement

The structure refinements were performed by full-matrix least-squares on F2. The H positions bound to C atoms were calculated after each cycle of refinement using a riding model C—H = 0.93 Å and *U*_iso_(H) = 1.2U_eq_(C). H atoms bound to N and O atoms were located in a difference Fourier map and refined in riding mode, U_iso_(H) = 1.2U_eq_(N) and 1.5U_eq_(O).

### Crystal data

**Table:**

C_8_H_9_N_4_S^+^·Cl^+^·H_2_O	*F*(000) = 512
*M**_r_* = 246.72	*D*_x_ = 1.472 Mg m^−^^3^
Monoclinic, *P*2_1_/*c*	Mo *K*α radiation, λ = 0.71073 Å
Hall symbol: -p 2ybc	Cell parameters from 5220 reflections
*a* = 9.3027 (6) Å	θ = 2.3–27.9°
*b* = 8.7038 (5) Å	µ = 0.51 mm^−^^1^
*c* = 14.5503 (8) Å	*T* = 293 K
β = 109.143 (3)°	Block, yellow
*V* = 1112.97 (11) Å^3^	0.33 × 0.19 × 0.14 mm
*Z* = 4	

### Data collection

**Table:**

Bruker SMART APEX CCD area-detector diffractometer	1958 independent reflections
Radiation source: fine-focus sealed tube	1818 reflections with *I* > 2σ(*I*)
Graphite monochromator	*R*_int_ = 0.021
ω scans	θ_max_ = 25.0°, θ_min_ = 2.3°
Absorption correction: multi-scan (*SADABS*; Bruker, 2001)	*h* = −11→11
*T*_min_ = 0.850, *T*_max_ = 0.932	*k* = −10→10
10318 measured reflections	*l* = −17→17

### Refinement

**Table:**

Refinement on *F*^2^	Primary atom site location: structure-invariant direct methods
Least-squares matrix: full	Secondary atom site location: difference Fourier map
*R*[*F*^2^ > 2σ(*F*^2^)] = 0.032	Hydrogen site location: inferred from neighbouring sites
*wR*(*F*^2^) = 0.083	H-atom parameters constrained
*S* = 1.07	*w* = 1/[σ^2^(*F*_o_^2^) + (0.041*P*)^2^ + 0.391*P*] where *P* = (*F*_o_^2^ + 2*F*_c_^2^)/3
1958 reflections	(Δ/σ)_max_ < 0.001
136 parameters	Δρ_max_ = 0.25 e Å^−^^3^
0 restraints	Δρ_min_ = −0.20 e Å^−^^3^

### Special details

**Table:**

Geometry. All e.s.d.'s (except the e.s.d. in the dihedral angle between two l.s. planes) are estimated using the full covariance matrix. The cell e.s.d.'s are taken into account individually in the estimation of e.s.d.'s in distances, angles and torsion angles; correlations between e.s.d.'s in cell parameters are only used when they are defined by crystal symmetry. An approximate (isotropic) treatment of cell e.s.d.'s is used for estimating e.s.d.'s involving l.s. planes.
Refinement. Refinement of *F*^2^ against ALL reflections. The weighted *R*-factor *wR* and goodness of fit *S* are based on *F*^2^, conventional *R*-factors *R* are based on *F*, with *F* set to zero for negative *F*^2^. The threshold expression of *F*^2^ > σ(*F*^2^) is used only for calculating *R*-factors(gt) *etc*. and is not relevant to the choice of reflections for refinement. *R*-factors based on *F*^2^ are statistically about twice as large as those based on *F*, and *R*- factors based on ALL data will be even larger.

### Fractional atomic coordinates and isotropic or equivalent isotropic displacement parameters (Å^2^)

**Table:**

	*x*	*y*	*z*	*U*_iso_*/*U*_eq_	
S1	0.37873 (6)	−0.13735 (5)	0.83964 (4)	0.05278 (17)	
N1	0.45104 (18)	0.12730 (17)	0.77931 (11)	0.0483 (4)	
H1N	0.4545	0.2261	0.7785	0.058*	
H2N	0.4887	0.0771	0.7417	0.058*	
N2	0.33167 (17)	0.15159 (16)	0.89088 (10)	0.0415 (3)	
H2	0.3490	0.2470	0.8830	0.050*	
N3	0.25238 (15)	−0.02567 (16)	0.99381 (10)	0.0372 (3)	
H3N	0.2705	−0.1020	0.9695	0.045*	
N4	0.22253 (15)	0.22080 (16)	1.00973 (10)	0.0394 (3)	
H4N	0.2267	0.3140	0.9987	0.047*	
C1	0.38850 (19)	0.0537 (2)	0.83586 (12)	0.0383 (4)	
C2	0.27105 (19)	0.11420 (19)	0.96112 (12)	0.0366 (4)	
C3	0.19110 (17)	−0.00866 (19)	1.06892 (11)	0.0348 (4)	
C4	0.1550 (2)	−0.1157 (2)	1.12841 (13)	0.0421 (4)	
H4	0.1668	−0.2207	1.1213	0.051*	
C5	0.1006 (2)	−0.0573 (2)	1.19897 (14)	0.0475 (4)	
H5	0.0780	−0.1248	1.2418	0.057*	
C6	0.0785 (2)	0.1001 (2)	1.20790 (14)	0.0487 (4)	
H6	0.0397	0.1343	1.2556	0.058*	
C7	0.11296 (19)	0.2059 (2)	1.14772 (13)	0.0445 (4)	
H7	0.0972	0.3106	1.1531	0.053*	
C8	0.17212 (18)	0.14874 (18)	1.07901 (12)	0.0360 (4)	
O1	0.44295 (16)	0.44905 (15)	0.86287 (10)	0.0522 (3)	
H1O	0.5262	0.4440	0.9042	0.078*	
H2O	0.3841	0.4980	0.8881	0.078*	
Cl1	0.23786 (6)	0.57314 (5)	0.98063 (4)	0.05321 (17)	

### Atomic displacement parameters (Å^2^)

**Table:**

	*U*^11^	*U*^22^	*U*^33^	*U*^12^	*U*^13^	*U*^23^
S1	0.0756 (4)	0.0303 (3)	0.0653 (3)	−0.0048 (2)	0.0406 (3)	−0.0053 (2)
N1	0.0621 (10)	0.0372 (8)	0.0539 (9)	−0.0004 (7)	0.0303 (8)	0.0037 (7)
N2	0.0569 (9)	0.0264 (7)	0.0446 (8)	−0.0004 (6)	0.0210 (7)	0.0028 (6)
N3	0.0462 (8)	0.0258 (7)	0.0404 (7)	0.0011 (6)	0.0152 (6)	−0.0025 (6)
N4	0.0479 (8)	0.0243 (7)	0.0456 (8)	0.0015 (6)	0.0150 (6)	0.0010 (6)
C1	0.0400 (9)	0.0361 (9)	0.0372 (8)	−0.0004 (7)	0.0104 (7)	0.0009 (7)
C2	0.0411 (9)	0.0288 (8)	0.0372 (8)	0.0000 (7)	0.0092 (7)	0.0006 (7)
C3	0.0347 (8)	0.0307 (8)	0.0371 (8)	0.0004 (6)	0.0094 (7)	−0.0016 (7)
C4	0.0455 (9)	0.0320 (9)	0.0489 (10)	0.0001 (7)	0.0156 (8)	0.0012 (7)
C5	0.0490 (10)	0.0459 (10)	0.0513 (11)	−0.0029 (8)	0.0214 (9)	0.0050 (8)
C6	0.0484 (10)	0.0511 (11)	0.0529 (11)	0.0017 (8)	0.0248 (9)	−0.0047 (9)
C7	0.0445 (10)	0.0356 (9)	0.0531 (10)	0.0049 (7)	0.0156 (8)	−0.0057 (8)
C8	0.0358 (8)	0.0313 (9)	0.0389 (9)	0.0001 (6)	0.0096 (7)	0.0001 (7)
O1	0.0616 (8)	0.0418 (7)	0.0558 (8)	0.0010 (6)	0.0227 (6)	−0.0045 (6)
Cl1	0.0654 (3)	0.0333 (3)	0.0646 (3)	0.0053 (2)	0.0264 (2)	0.0047 (2)

### Geometric parameters (Å, º)

**Table:**

S1—C1	1.6673 (18)	C3—C4	1.386 (2)
N1—C1	1.319 (2)	C3—C8	1.395 (2)
N1—H1N	0.8607	C4—C5	1.382 (3)
N1—H2N	0.8592	C4—H4	0.9300
N2—C2	1.359 (2)	C5—C6	1.397 (3)
N2—C1	1.387 (2)	C5—H5	0.9300
N2—H2	0.8606	C6—C7	1.379 (3)
N3—C2	1.339 (2)	C6—H6	0.9300
N3—C3	1.396 (2)	C7—C8	1.382 (2)
N3—H3N	0.7964	C7—H7	0.9300
N4—C2	1.332 (2)	O1—H1O	0.8112
N4—C8	1.393 (2)	O1—H2O	0.8651
N4—H4N	0.8304		
			
C1—N1—H1N	121.3	C4—C3—N3	131.46 (15)
C1—N1—H2N	120.4	C8—C3—N3	106.54 (14)
H1N—N1—H2N	118.4	C5—C4—C3	116.06 (16)
C2—N2—C1	128.15 (14)	C5—C4—H4	122.0
C2—N2—H2	118.3	C3—C4—H4	122.0
C1—N2—H2	113.1	C4—C5—C6	122.05 (18)
C2—N3—C3	108.39 (14)	C4—C5—H5	119.0
C2—N3—H3N	122.0	C6—C5—H5	119.0
C3—N3—H3N	129.5	C7—C6—C5	121.59 (17)
C2—N4—C8	108.90 (13)	C7—C6—H6	119.2
C2—N4—H4N	122.2	C5—C6—H6	119.2
C8—N4—H4N	128.9	C6—C7—C8	116.69 (17)
N1—C1—N2	113.04 (15)	C6—C7—H7	121.7
N1—C1—S1	123.02 (14)	C8—C7—H7	121.7
N2—C1—S1	123.94 (13)	C7—C8—N4	132.05 (15)
N4—C2—N3	109.79 (15)	C7—C8—C3	121.58 (16)
N4—C2—N2	121.94 (15)	N4—C8—C3	106.37 (14)
N3—C2—N2	128.26 (15)	H1O—O1—H2O	107.1
C4—C3—C8	121.98 (16)		
			
C2—N2—C1—N1	−174.63 (16)	C3—C4—C5—C6	2.0 (3)
C2—N2—C1—S1	5.7 (3)	C4—C5—C6—C7	−1.3 (3)
C8—N4—C2—N3	1.18 (19)	C5—C6—C7—C8	−0.8 (3)
C8—N4—C2—N2	−177.76 (15)	C6—C7—C8—N4	−177.98 (17)
C3—N3—C2—N4	−1.16 (18)	C6—C7—C8—C3	2.1 (2)
C3—N3—C2—N2	177.68 (16)	C2—N4—C8—C7	179.38 (17)
C1—N2—C2—N4	178.93 (15)	C2—N4—C8—C3	−0.71 (18)
C1—N2—C2—N3	0.2 (3)	C4—C3—C8—C7	−1.5 (3)
C2—N3—C3—C4	−177.73 (17)	N3—C3—C8—C7	179.93 (15)
C2—N3—C3—C8	0.69 (18)	C4—C3—C8—N4	178.62 (15)
C8—C3—C4—C5	−0.6 (2)	N3—C3—C8—N4	0.01 (17)
N3—C3—C4—C5	177.59 (16)		

### Hydrogen-bond geometry (Å, º)

**Table:**

*D*—H···*A*	*D*—H	H···*A*	*D*···*A*	*D*—H···*A*
O1—H1*O*···Cl1^i^	0.81	2.29	3.0991 (15)	179
O1—H2*O*···Cl1	0.87	2.30	3.1443 (14)	165
N1—H1*N*···O1	0.86	2.32	3.064 (2)	145
N1—H2*N*···O1^ii^	0.86	2.14	3.000 (2)	174
N2—H2···O1	0.86	2.03	2.8667 (19)	165
N3—H3*N*···S1	0.80	2.43	3.0146 (14)	131
N4—H4*N*···Cl1	0.83	2.28	3.1055 (15)	175

Symmetry codes: (i) −*x*+1, −*y*+1, −*z*+2; (ii) −*x*+1, *y*−1/2, −*z*+3/2.
